# A real-world study on diagnosis and prognosis of light-chain cardiac amyloidosis in Southern China

**DOI:** 10.1186/s12872-021-02256-3

**Published:** 2021-09-18

**Authors:** Zhijian Wu, Muzheng Li, Tudahun Ilyas, Wei Li, Mu Zeng, Fang Li, Yanxia Liu, Mingxian Chen, Yaqin Chen, Qingyi Zhu, Nenghua Qi, Qiming Liu, Jianjun Tang

**Affiliations:** 1 Department of Cardiovascular Medicine, The Second Xiangya Hospital of Central South University, No. 139 Middle Renmin Road, Furong District, Changsha City, 410011 Hunan Province China; 2 Department of Cardiology, Huaihua Hospital of Traditional Chinese Medicine, Huaihua, 418000 China; 3 Department of Radiology, The Second Xiangya Hospital of Central South University, Changsha, 410011 China; 4 Department of Radiology, Zhuzhou Central Hospital, Zhuzhou, 412000 China

**Keywords:** Cardiac amyloidosis, Light chain, Heart failure, Prognosis, Real-world study

## Abstract

**Background:**

Light-chain cardiac amyloidosis (AL-CA) has been highly valued in developed countries, but in developing countries, the recognition and diagnosis of this condition is still limited. There are currently few reports on a large number of Chinese patients with AL-CA. The present study aimed to report real-world clinical characteristics and prognosis of AL-CA in China.

**Methods and results:**

Consecutive patients with AL-CA diagnosed at the Second Xiangya Hospital of Central South University between June 2012 and September 2020 were reviewed. A total of 170 patients with AL-CA have been recruited, whose mean ages were 60.81 ± 10.46. 70.59% of the patients were male. They were from eight provinces in southern China, 55.7% were referred patients, and 37.3% had been misdiagnosed previously. 64 (37.6%) patients received chemotherapy. The median survival time for patients with AL-CA was 8.00 months, and survival time for patients who received chemotherapy was 13.00 months, which was significantly longer than that of patients with palliative treatment (13.00 vs 6.00, *p* = 0.004).

**Conclusions:**

Although clinicians have improved their understanding of AL-CA in recent years, the prognosis of AL-CA is still poor, and the misdiagnosis rate and missed diagnosis rate are still very high in China. It is imperative to improve the recognition and early diagnosis of this condition, which may require multidisciplinary collaboration among cardiologists, hematologists and nephrologists.

**Supplementary Information:**

The online version contains supplementary material available at 10.1186/s12872-021-02256-3.

## Introduction

Cardiac amyloidosis (CA) is a rare condition in which the myocardial interstation is expanded by an amorphous, β-sheet structured fibrillar proteinaceous material known as amyloid, resulting in progressive injury of cardiomyocytes and eventually leading to arrhythmia and heart failure [1, 2]. Although more than ten kinds of amyloid proteins can involve the heart, light chain CA (AL-CA) and transthyretin CA (TTR-CA) are the most two common types in clinical practice [3]. TTR-CA is classified by the sequence of the TTR gene, either wild-type transthyretin amyloid CA (ATTRwt-CA) (no mutation) or mutant transthyretin amyloid CA (ATTRm-CA) (a mutation is present). CA often manifests as diffuse myocardial hypertrophy and heart failure with preserved ejection fraction (HFpEF). It should be emphasized that it has a very poor prognosis compared with other types of cardiomyopathy [4]. Especially for AL-CA, the natural course of untreated AL-CA is about 6 months [5]. Developed countries such as the United States, some European countries and Japan have published some high-quality articles on the clinical characteristics and epidemiological investigation of AL-CA, which is of great significance to promote the diagnosis and treatment of CA [6, 7]. Enormous advances have been made over the last decade, both in the diagnosis and treatment of AL-CA, along with a recognition that the condition is more common than previously believed.

However, in developing countries such as China, due to the complex clinical manifestations and limited diagnostic and therapeutic methods, most cardiologists do not know enough about AL-CA, and many patients with AL-CA are misdiagnosed and missed. At the time of definite diagnosis, most of the patients were already in the late stage of the disease and lost the opportunity of early treatment [8, 9]. These result in a worse prognosis for AL-CA patients in China compared with developed countries. Meanwhile, a large number of case reports on the clinical characteristics and real-world prognosis of AL-CA are very few in China. Therefore, to strengthen the understanding and improve the level of diagnosis and treatment of AL-CA has become an urgent task in the field of cardiovascular medicine in China [10].

The purpose of the present study was to describe the main clinical characteristics, diagnostic approach and prognosis of a large cohort of patients with AL-CA in China.

## Methods

### Patients

We enrolled 170 consecutive patients diagnosed with AL-CA at the Second Xiangya Hospital of Central South University between June 2012 and September 2020. Data including demographic characteristics, comorbidities, first main complaint at diagnosis, laboratory testing results, electrocardiographic (ECG), echocardiographic data, imaging, histopathology findings and treatment were obtained.

The study protocol conformed to the ethical guidelines of the Declaration of Helsinki [11] as reflected by prior approval from the human research committee of the Second Xiangya Hospital of Central South University. Written informed consent was obtained from patients while the patient was in a clinically stable, non-congested condition or their family members who can give informed consent on behalf of patients after they were informed about the objectives and procedures of the study. Their rights to refuse participation any time they want were assured. For this purpose, a one-page consent letter was attached as a cover page of each questionnaire stating the general objective of the study and issues of confidentiality that was discussed by the data collectors before proceeding to the data collection.

### Data collection

Demographic and clinical characteristics were collected on admission. Recording the clinical features that led to the diagnosis of CA and the previous diagnosis, history of previous visits according to the functional classification data of the New York Heart Association (NYHA). Comorbidities were listed according to what the patient told our doctor on admission and what we diagnosed after discharge.

Blood test parameters were collected from the first blood test available. Cockcroft-Gault formula was used for the rough calculation of estimated glomerular filtration rate (eGFR). Body mass index (BMI), calculated as weight divided by height squared (kg/m^2^). Subclinical hypothyroidism is defined as no clinical symptoms, normal serum FT3 and FT4, and serum TSH concentration exceeding the reference value.

### ECG

A standard 12-lead ECG recording (GE Healthcare, filter range, 50 Hz, 25 mm/s, 10 mm/mV) was part of standard evaluation at the time of patients admission to hospital. The presence of a complete left or right bundle branch block was defined according to standard published criteria [12, 13]. QTc was calculated based on Bazett's formula. Low voltage was defined as a QRS amplitude ≤ 0.5 mV in all limb leads or ≤ 1.0 mV in all precordial leads. A pseudo infarction pattern was defined as pathological Q waves or QS waves on 2 consecutive leads in the absence of previous ischemic heart disease. Poor R-wave progression (PRWP) was defined as RV3 ≤ 3 mm and RV2 ≤ RV3.

### Diagnostic procedures

The diagnostic criteria for suspected CA are symptoms of HF, echocardiography indicated that the interventricular septum and the posterior thickness of left ventricular are ≥ 12 mm; ECG indicated low voltage in the limb leads, positive serum free light chain or blood/urine Bence Jones protein. Then, patients will perform some non-invasive or tissue biopsies in order to confirm the diagnosis.

The diagnosis of AL amyloidosis was confirmed based on previous literature reports [1] and described below 1.positive serum free light chain or blood/urine Bence Jones protein; 2.the presence of apple-green appearance viewed under cross-polarized light with Congo red staining and tissue typing by immunohistochemistry on tissue biopsies from endocardial myocardial tissue or at least one clinically involved organ, including abdominal fat tissue, bone marrow, kidney, intestinal mucosa; 3.a typical diffuse subendocardial or transmural late gadolinium enhancement pattern on cardiac magnetic resonance (CMR). Patients compliance with 1 + 2 or 1 + 3 was included in this study.

### Follow-up and prognosis

Follow-up started at the time of diagnosis of AL-CA. The primary endpoint for this study was death from any cause. Survival time (months) was measured as the duration between the first day of hospitalization when the patient was diagnosed with AL-CA to the date of death; if it exceeded 15 days, it was calculated as 1 month. Data were obtained from medical records or from telephone interviews with patients or relatives by 4 trained physicians. Patients were followed until November 16, 2020. Patients were censored if they were still alive at the end of the research period or were lost to follow-up, on which occasion their last clinic visit or correspondence time was used.

### Statistical analysis

Normally distributed parameters were expressed as mean ± standard deviation (SD), whereas non-normally distributed parameters were expressed as median with inter-quartile range (Q3–Q1). Categorical values were presented as numbers (percentages). Categorical data were reported as frequencies and percentages and were compared using Chi-squared or Fisher’s exact test. Comparison of continuous variables between two independent groups was performed using unpaired Student’s t-test (if normally distributed) or Mann–Whitney U-test (non-normally distributed variables) and in cases where more than two groups were compared, one-way analysis of variance (ANOVA) or Kruskal–Wallis test was used. Survival was evaluated with Kaplan–Meier curves. All tests were 2-tailed and a *p* value of < 0.05 was considered to be statistically significant. Statistical analysis was performed using SPSS 26.0 (IBM Software Inc), EmpowerStats 3.0 software and R (version 3.3.2).

## Results

### Clinical features

#### Demographic characteristics

During the study period, a total of 170 patients were diagnosed with AL-CA. The mean age at diagnosis was 60.81 ± 10.46. 70.59% (120/170) of the patients were male. They were from eight provinces in southern China (more details were shown in Additional files 1, 2, 3, 4), 55.7% were referred patients, and 37.3% had been misdiagnosed previously.

#### Clinical profile leading to diagnosis

**Fig. 1:**
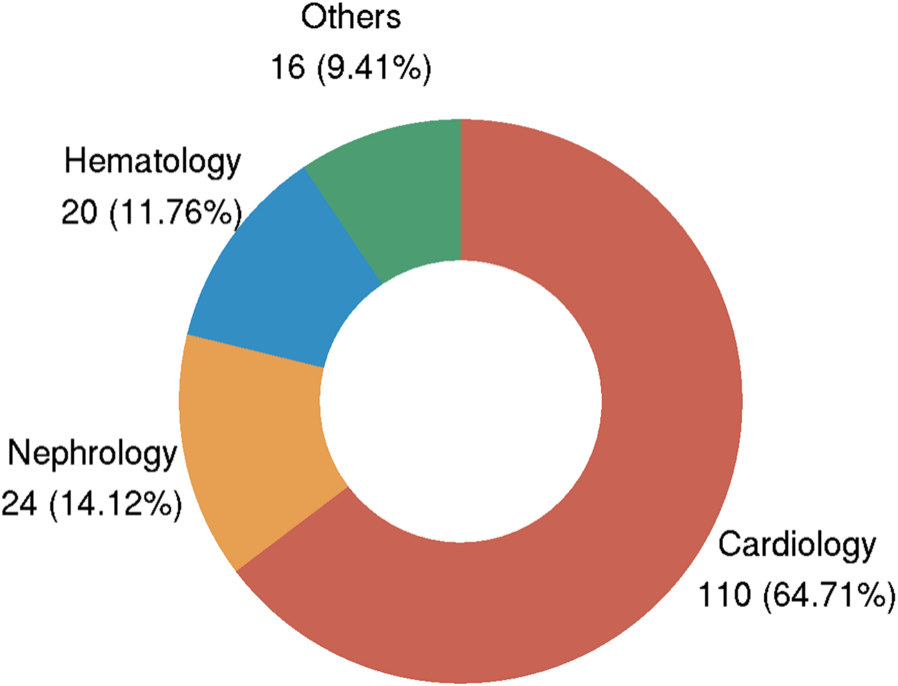
The department of patients with AL-CA first visited and were confirmed

Heart failure was the first chief complaint in the majority of patients, 52.94% presented with dyspnea (chest tightness, shortness of breath, orthopnea, nocturnal paroxysmal dyspnea, etc.) and 22.35% of patients presented with edema of lower limbs. 76.47% of patients presented with NYHA III-IV. 70.7% of the patients were classified as grade III according to the Mayo AL 2004 stage system, and only 4% of patients were classified as grade I. Most of the patients were diagnosed in the Department of Cardiology, 24 (14.12%) patients with AL-CA were diagnosed in department of nephrology Fig. 1.

#### Comorbidities

Regarding comorbidities, 58 (34.12%) patients of AL-CA were diagnosed with multiple myeloma (MM). 115(67.65%) patients presented with polyserositis and 44 (25.88%) patients had subclinical or clinical hypothyroidism. 58 (34.12%) patients were previously diagnosed with hypertension, 35(20.59%) patients had history of CHD and 41 (24.12%) had hyperlipidemia. The remaining comorbidities are shown in Table1.

**Table 1:** Clinical characteristics of 170 patients with AL-CA in this study

	AL-CA (n = 170)
Male, n (%)	120 (70.59%)
Age at diagnosis, years	60.81 (10.46)
Previous misdiagnosis, n (%)	56 (32.9%)
Patients of referred, n (%)	92 (54.1%)
Length of hospital stay, days	15.94 (9.35)
*First chief complaint, n (%)*	
Dyspnea	90 (52.94%)
Edema in lower limbs	38 (22.35%)
Fatigue	14 (8.24%)
Others	28 (16.47%)
*Department of first diagnosis, n (%)*	
Cardiology	110 (64.71%)
Nephrology	24 (14.12%)
Hematology	20 (11.76%)
Others	16 (9.41%)
*NYHA, n (%)*	
I	7 (4.12%)
II	33 (19.41%)
III	74 (43.53%)
IV	56 (32.94%)
*Mayo AL 2004 stage, n (%)*	
I	6(4.0%)
II	38(25.3%)
III^a^	51(34%)
III^b^	55(36.7%)
*Comorbidities, n (%)*	
Multiple myeloma	58 (34.12%)
Polyserositis	115 (67.65%)
CHD	35(20.59%)
Subclinical or clinical hypothyroidism	44 (25.88%)
Peripheral neuropathy	46(27.1%)
Previous hypertension	58 (34.12%)
Hyperlipidemia	41 (24.12%)
Type 2 diabetes	22 (12.94%)
COPD	19 (11.18%)
CKD	56 (32.94%)
Stroke	12 (7.06%)
SBP, mmHg	114.23 (24.18)
DBP, mmHg	71.18 (12.50)
Pulse, Times/min	82.41 (17.82)
BMI, kg /m^2^	22.5 (3.5)
Death in hospital, n (%)	21 (12.4%)
*Assistant examination methods, n (%)*	
Non-invasive, n (%)	
CMR	88 (51.7%)
SPECT	17 (10.0%)
*Biopsy, n (%)*	
Endocardial	21 (12.3%)
Bone marrow	134(78.9%)
Renal	13(7.6%)
Subcutaneous fat	89(52.4%)
Gastrointestinal	7(4.1%)
Others^a^	2(1.2%)
*Treatment*	
Pacemaker	14 (8.2%)
ACEI/ARB	40 (23.5%)
Beta-blockers	35 (20.6%)
Loop diuretics	154 (90.6%)
Antisterone	127 (74.7%)
Tolvaptan	15 (8.8%)
LMWH	29 (17.1%)
Warfarin	12 (7.1%)
Digitalis	32 (18.8%)
Aspirin	38 (22.4%)
Clopidogrel	28 (16.5%)
Statins	62 (36.5%)
*Chemotherapy, n (%)*	64 (37.6%)
B + D	39 (22.9%)
Melphalan-based	7 (4.1%)
ThD/LeD	56 (32.9%)
ASCT	2(1.2%)
Palliative care, n (%)	106(62.4%)

Date are shown as (N) Mean (SD) or n (percentage)

NYHA: New York heart association functional classification; CHD: coronary heart disease; COPD: chronic obstructive pulmonary disease; CKD: chronic kidney disease; SBP: systolic blood pressure; DBP: diastolic blood pressure; BMI: body mass index. ECG: electrocardiographic; CMR: cardiac magnetic resonance; LMWH: low molecular weight heparin; B + D: bortezomib + dexamethasone, ASCT: autologous stem cell transplantation; ThD/LeD: thalidomide/lenalidomide

^a^Tongue biopsy was performed in 2 patients with AL-CA

#### Diagnostic approach and treatment

The diagnostic approach and treatment were illustrated in Table 1. CMR was performed in 88 (51.7%) patients. As for biopsy, 134(78.9%) cases underwent bone marrow biopsy and 89(52.4%) cases underwent subcutaneous fat biopsy. Endocardial biopsy was performed in 21 (12.3%) cases.

In terms of medical therapy, 40 (23.5%) AL-CA cases were received ACEI or ARB. 35 (20.6%) AL-CA cases were on beta-blockers. 64 (37.6%) AL-CA patients received chemotherapy, of which 39 (22.9%) received bortezomib + dexamethasone. 56 (32.9%) patients were received thalidomide / lenalidomide. 14 (8.2%) AL-CA cases were treated with a pacemaker. More than 90% of the patients were prescribed furosemide during hospitalization.

#### Laboratory, echocardiographic and electrocardiographic characteristics

Laboratory test results are shown in Table 2.

**Table 2:** Laboratory, echocardiographic and electrocardiographic characteristics in patients with AL-CA in the present study

	Normal range	AL-CA (n = 170)
*Laboratory results*		
Hemoglobin, g/L	115–150	113.6 (22.7)
White blood cell,10^9/L	3.5–9.5	6.6 (3.0)
Platelets, 10^9^/L	125–350	191.4 (90.7)
Potassium, mmol/L	3.5–5.3	4.1 (0.5)
Calcium, mmol/L	2.11–2.52	2.1 (0.2)
ALT, u/L	7.0–40	19.6 (13.4–28.3)
AST, u/L	13.0–35.0	27.5 (20.3–36.3)
Albumin, g/L	40.0–55.0	29.9 (6.8)
eGFR, mL/(min × 1.73m^2^)	90–120	60.6 (31.4)
Uric acid, umol/L	155–357	455.5 (168.5)
24-h urine protein ≥ 1.0 g, n%	–	90 (53.0%)
Cardiac troponin T, pg/ml	0–14	84.8 (36.4–158.8)
CK-MB, u/L	0–24	14.6 (10.2–18.9)
NT-proBNP, pg/mL	100–300	6139.4 (2957.7–11,754.0)
LDH, U/L	120–250	268.6 (216.8–331.5)
CRP, mg/L	0–8	22.6 (39.0)
TC, mmol/L	2.9–5.2	4.1 (2.1)
LDL-c, mmol/L	< 3.12	2.6 (1.4)
D-Dimer, ug/mL	0–0.55	1.7 (1.8)
*Echocardiographic findings*		
LVEDd, mm	Male: ≤ 55, Female ≤ 50	44.0 (7.1)
RVEDd, mm	≤ 35	32.5 (4.9)
LAESd, mm	Male: ≤ 35, Female ≤ 30	41.0 (7.3)
RAESd, mm	≤ 30	38.4 (8.5)
IVS, mm	6–11	14.1 (3.6)
LVPW, mm	6–11	13.7 (3.3)
LVEF, (%)	> 50%	52.9 (10.0)
*ECG findings*		
Atrial fibrillation, n%	–	(164) 29 (17.7%)
AV block grade I or greater, n%	–	(164) 23 (14.0%)
Low voltage, n%	–	(163) 93 (57.1%)
Pseudo necrosis, n%	–	(163) 6 (3.7%)
PRWP, n%	–	(163) 138 (84.7%)
PR interval, ms	120–200	(133) 173.8 (40.8)
Corrected QT interval, ms	320–440	(163) 471.6 (36.7)
QRS duration, ms	60–110	(163) 101.6 (38.0)
Ventricular rate, n/min	60–100	(164) 81.9 (19.6)

Data are (N) Mean (SD) or (N) n (%), Median (Q3–Q1), where N is the total number of patients with available data

ALT: Alanine transaminase; AST: aspartate aminotransferase; eGFR: estimated glomerular filtration rate; CK-MB: creatine kinase myocardial isoenzyme; CRP: C-reactive protein; LDL-C: low density lipoprotein-cholesterol; TC: total cholesterol; LDH: lactate dehydrogenase; NT-proBNP: N-terminal pro–B-type natriuretic peptide; LVEDd: left ventricular end diastolic diameter; RVEDd: right ventricular end diastolic diameter; LAESd: left atrium end systolic diameter; RAEDd: right atrium end systolic diameter; IVS: interventricular septum; LVPW: left ventricular posterior wall; LVEF: left ventricular ejection fraction; PRWP: poor R-wave progression

Mean LVEF of AL-CA cases were 52.9 ± 10.0%. 65 patients (65/169) with AL-CA showing a LVEF < 50%. AL-CA cases had normal left and right ventricular end diastolic diameter and enlarged atrial end systolic diameter. In addition, the right atrial end systolic diameter was larger than the right ventricular end diastolic diameter (Table 2). 29 AL-CA (17.7%) had any form of atrial fibrillation (AF). 63.2% AL-CA cases fulfilled low QRS voltage according to limb and precordial leads criteria. Other ECG findings are shown in Table 2.

#### Univariate and multivariate predictors of all-cause mortality

We investigated the prognostic factors for all-cause mortality using univariate and multivariate Cox hazard analyses in our study, as presented in Table 3. In the univariate model, systolic blood pressure (HR 0.988, 95%CI 0.981–0.996, *p* = 0.002), diastolic blood pressure (HR 0.983, 95%CI 0.969–0.996, *p* = 0.011), NT-proBNP (HR 1.000, 95%CI 1.000–1.000, *p* = 0.001), hemoglobin (HR 1.008, 95%CI 1.000–1.017, *p* = 0.041),chemotherapy (HR 0.571, 95%CI 0.382–0.853, *p* = 0.006), D-Dimer (HR 1.138, 95%CI 1.046–1.237, *p* = 0.003), interventricular septum (HR 1.089, 95%CI 1.037–1.143, *p* = 0.001), LVEF (HR 0.971, 95%CI 0.952–0.990, *p* = 0.003),left ventricular posterior wall (HR 1.084, 95%CI 1.023–1.149, *p* = 0.007), and low voltage (HR 1.572, 95%CI 1.061–2.331, *p* = 0.024) were statistically significant predictors of overall survival, however, when multivariate analysis was performed, there were only two factors remained as determinants, LVEF (HR 0.965, 95%CI 0.942–0.988, *p* = 0.003) and interventricular septum (HR 1.191, 95%CI 1.033–1.373, *p* = 0.016).

**Table 3:** Univariate and multivariate cox hazard analyses of predictors for all-cause mortality

Variables	Univariate			Multivariate		
	HR	95% CI	*p* value	HR	95% CI	*p* value
Male	0.890	0.596–1.328	0.568	–	–	–
Age	1.009	0.992–1.026	0.312	–	–	–
NYHA	1.099	0.999–1.210	0.052	–	–	–
SBP	0.988	0.981–0.996	**0.002**	0.995	0.980–1.011	0.561
DBP	0.983	0.969–0.996	**0.011**	0.988	0.959–1.018	0.424
MM	1.180	0.799–1.742	0.405	–	–	–
Hyperlipidemia	0.847	0.555–1.294	0.443	–	–	–
Polyserositis	1.273	0.856–1.894	0.233	–	–	–
Hemoglobin	0.992	0.983–1.000	**0.041**	0.999	0.988–1.010	0.860
Atrial fibrillation	1.047	0.656–1.673	0.847			
ALB	0.991	0.963–1.020	0.535	–	–	–
Calcium	1.145	0.505–2.598	0.746	–	–	–
eGFR	0.990	0.988–1.004	0.372	–	–	–
Cardiac troponin T	1.001	1.000–1.002	0.134	–	–	–
NT-proBNP	1.000	1.000–1.000	**0.001**	1.000	1.000–1.000	0.065
Chemotherapy	0.571	0.382–0.853	**0.006**	0.803	0.481–1.341	0.402
LDL-C	1.148	0.992–1.329	0.065	–	–	–
TC	1.096	0.988–1.214	0.082	–	–	–
D-Dimer	1.138	1.046–1.237	**0.003**	1.085	0.980–1.201	0.116
IVS	1.089	1.037–1.143	**0.001**	1.191	1.033–1.373	**0.016**
LVPW	1.084	1.023–1.149	**0.007**	0.911	0.785–1.058	0.222
LVEF	0.971	0.952–0.990	**0.003**	0.965	0.942–0.988	**0.003**
Low voltage	1.572	1.061–2.331	**0.024**	1.118	0.702–1.780	0.638
PRWP	0.238	0.416–1.243	0.238	–	–	–
QRS duration	1.001	0.994–1.007	0.865	–	–	–

HR, hazard ratio; CI: Confidence Interval; p value in bold is considered statistically significant

For other abbreviations, see Table 1

#### Patient survival

**Fig. 2:**
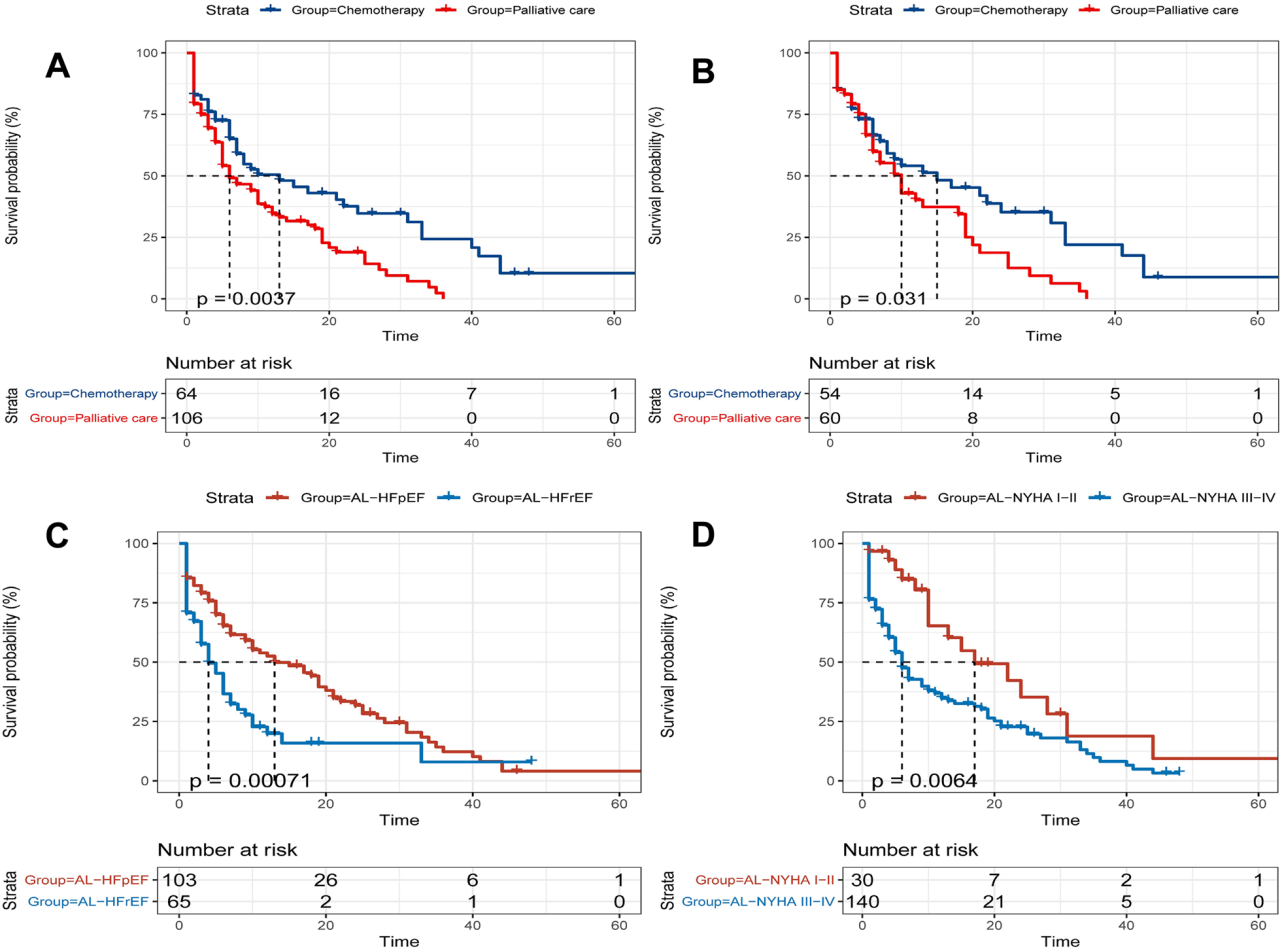
Kaplan–Meier survival curves demonstrating differences in overall survival (months). **a** The median survival time of Al-CA patients receiving chemotherapy was significantly longer than that of patients receiving palliative treatment (13.00 vs 6.00, log-rank test, *p* = 0.004). **b** In patients with NYHAI-III, receiving chemotherapy improved outcome significantly (15.00 vs 10.00, *p* = 0.031). **c** In AL-CA group, survival time was significantly higher in patients who were HFpEF (LVEF < 50%) (13.00 vs 4.00, *p* = 0.001). (D)Median survival time of patients with NYHA III-IV was shorter than NYHA I-II group (6.00 vs 17.00, *p* = 0.006)

The median follow-up period was 5.0 (range, 1.0– 17.0) months in AL-CA group, and 117(68.82%) patients died during the observation period. In AL-CA group, the median survival time of AL-CA patients receiving chemotherapy was 13.00 months, which was significantly longer than that of patients receiving palliative treatment (13.00 vs 6.00, *p* = 0.004) (Fig. 2a). In patients with NYHA I-III, receiving chemotherapy improved outcome significantly (15.00 vs 10.00, *p* = 0.031) (Fig. 2b). In AL-CA group, survival time was significantly higher in patients who were HFpEF (LVEF ≥ 50%) (13.00 vs 4.00, *p* = 0.001) (Fig. 2c). Median survival time of patients with NYHA III-IV was shorter than NYHA I-II group (6.00 vs 17.00, *p* = 0.006). (Fig. 2d) Other median survival time of each subgroup are shown in Additional files 1, 2, 3, 4.

## Discussion

This 8-year retrospective cohort study, conducted in a large tertiary referral teaching hospital in southern China, describes the current situation and provides insights on diagnosis, treatment and survival of AL-CA in China. To our knowledge, this is the largest number of cases report to evaluate the characteristics and the prognosis of patients with AL-CA in southern China.

AL-CA is a hematologic disorder of plasma cells closely related to, and more commonly diagnosed by Chinese cardiovascular physicians compared with TTR-CA. Several decades ago, some reports from Europe [6] and USA [14] systematically revealed the clinical characteristics and prognosis data of this condition. Compared with previous reports from developed countries, the demographic characteristics for AL-CA patients seem to be similar. Dubrey et al. [14] reported a nationwide survey on AL-CA from USA (n = 232), and revealed the majority of patients with AL-CA were male (n = 142, 61%) with a mean (± SD) age at diagnosis of 60 ± 11 years. In our AL-CA cases group, mean ages were 60.81 ± 10.46 and 70.59% (120/170) were male. Previous studies [6, 15] showed that the major manifestation leading to diagnosis was HF (80%), which was confirmed in 72.3% of this study subjects. (Table 4) The department of cardiovascular medicine was not the only department to diagnose AL-CA patients in the present study, 24 patients (14.12%) of AL-CA patients were diagnosed in nephrology, and 20 patients (11.76%) were diagnosed in hematology (Fig. 1).

**Table 4:** Clinical characteristics and prognosis in the present study compared with previous studies in some developed countries

	Present study (n = 170)	Pinney et al. [5] (n = 36)	Sidana [6] (n = 405)	Quarta et al. [14] (n = 80)
Country	Changsha, China	London, UK	Rochester, USA	Boston, USA
Age at diagnosis (years)	60.81 (± 10.46)	63.0 (56.6–65.8)	65 (58–73)	62 (± 10)
Male (%)	70.59%	69%	64%	66%
Major manifestation leading diagnosis (%)	HF (72.3%)	HF (80.5%)	NA	HF (66%)
Pacemaker	8.2%	5.5%	NA	NA
Atrial fibrillation	17.7%	11%	NA	8%
NYHA III or IV (%)	76.47%	60%	NA	29%
eGFR, mL/(min × 1.73m^2^)	60.6 (± 31.4)	64 (48–87)	62 (46–77)	64 (± 27)
NT-proBNP, pg/mL	6139.4 (2957.7–11,754.0)	714.0 (427.5–1573.0) pmol/L	4484 (1846–10 243)	3085 (1314–11,260)
IVS, mm	14.1 (± 3.6)	15 (± 2)	NA	15 (± 2)
LVPW, mm	13.6 (± 3.3)	15 (± 2)	NA	14 (± 2)
LVEF, (%)	52.9 (± 10.0)	47.8 (± 12.6)	NA	56 (± 14)
QTc, ms	471.6 (± 36.7)	596.6 (± 745.0)	NA	NA
Low voltage	57.1%	27%	NA	45%
Median survival (month)	8.0	10.4	16	12

Data are (N) Mean (SD) or (N) n (%), Median (Q3–Q1), where N is the total number of patients with available data. For other abbreviations, see Tables 1, 2 and 3

In terms of assistant examination methods, extracardiac tissue biopsy was the most common assistant diagnostic procedure, with bone marrow biopsy performed in 134 (78.9%) AL-CA patients and subcutaneous fat biopsy performed in 89(52.4%) AL-CA patients, and CMR only performed in 88 (51.7%) patients. Endomyocardial biopsy was performed in only 21 (12.3%) patients. Since extracardiac biopsies are often associated with a high proportion of false negatives, some current studies [16–19] have found CMR to have a high sensitivity and specificity for diagnosing AL-CA patients or even superior to extracardiac biopsies, but many patients cannot afford it due to the high price. Histological confirmation of endocardial biopsy is the gold standard for the diagnosis of suspected CA [7]. However, only a small number of patients rely on this method for diagnosis. This is different from previous published studies from developed countries, where the proportion of diagnosis based on endocardial biopsy is generally more than 50%. In addition to the lack of skilled technicians and the high price, a large proportion of patients had poor cardiac function, could not lie flat, and could not tolerate CMR and endomyocardial biopsy.

In our study, the median survival time from diagnosis to death was 8 months in the AL-CA group. This is the same as 0.75 years (9 months) reported by Dubrey et al. [14] in the USA 20 years ago. Recently (published in 2019), Sidana et al. [20] reported a median survival of 16 months in patients with AL-CA in the current USA. The prognosis of AL-CA patients in China have a worse prognosis compared with those living in the USA or Europe. This may be attributed to the following reasons after comparing the previous studies. Firstly, when AL-CA was diagnosed, most of the patients were in severe condition. In our study, 76.5% (130/170) of the patients were NYHA III-IV. In the study of Quarta et al. [15], only 29% of the patients were NYHA III-IV. Secondly, the proportion of patients receiving chemotherapy is small. In the cohort of Sidana et al. [20], among 348 patients, 60 (17%) patients receiving autologous stem cell transplantation (ASCT), 83 (24%) patients receiving bortezomib, 144 (41%) patients receiving alkylator and/or immunomodulatory drugs and only 54 (16%) patients receiving palliative care. However, 106 (62.4%) patients in our study were receiving palliative care, and in chemotherapy group, only 39 (22.9%) patients receiving bortezomib. According to previous studies. [21, 22] and data from the present study, patients receiving chemotherapy live significantly longer. Besides, in the real world, most of the patients in the chemotherapy group in our study had a cardiac function grade of NYHA I-III, which may lead to a bias of the data. So, we did subgroup analysis and found that, in patients with NYHA I-III, receiving chemotherapy equally improved outcome significantly (15.00 vs 10.00, *p* = 0.031) (Fig. 2b). This illustrates that the proportion of chemotherapy received is also an important reason affecting the prognosis of AL-CA patients. There is also a phenomenon to point out that some patients who choose palliative treatment use thalidomide or lenalidomide monotherapy in our study. Whether these people actually benefit from it is not clear.

In this study, we also reveal some urgent clinical problems in the diagnosis and treatment of AL-CA in China. First of all, the misdiagnosis and missed diagnosis rate of AL-CA was high, 56 (32.9%) patients with AL-CA were misdiagnosed in the past. This shows that cardiovascular physicians in primary hospitals in China have insufficient knowledge of this kind of disease. According to previous studies [23, 24] in European and American countries, approximately 10% of patients with AL-CA will have evidence of overt multiple myeloma(MM), and a similar proportion of MM patients will have AL-CA. However, 58 (34.12%) were multiple myeloma in our study, a proportion much higher than that in the countries mentioned above. We therefore speculate that there are a significant number of patients with MM in whom cardiac involvement has been overlooked. Of course, we also cannot rule out an ethnic distinction in AL-CA, therefore, it is necessary to strengthen the understanding and diagnosis of AL-CA in China and more studies are needed to report the clinical features of AL-CA in Chinese, especially the clinical features and natural history. Secondly, the prevalence of non-invasive diagnosis of CA is low. In recent years, some studies [18, 19] have revealed that CMR has strong specificity in the diagnosis of AL-CA. These are powerful noninvasive diagnostic methods to assist in the diagnosis of AL-CA and can make up for the false negative situation of tissue biopsy. However, the utilization rate of CMR in China is at a low level. Thirdly, AL-CA patients did not receive timely chemotherapy, most physicians abandoned to prescribe chemotherapy based on uncertainty about the clinical diagnosis and many patients needed to be diagnosed with MM before they could be administered chemotherapy, which resulted in patients losing the opportunity for early treatment in the early course of their disease. Of course, increasing the rate of treatment also requires to improve clinical diagnostic accuracy, which may need to benefit from clinically experienced physicians and noninvasive diagnostic methods such as CMR.

## Limitations

Although our study tries to truly reflect the diagnosis and treatment status of AL-CA in China, it is just a single center retrospective cohort study. In this study, only a small number of AL-CA patients were confirmed by endocardial biopsy, and most of them were confirmed by non-myocardial biopsy histological confirmation and CMR. For follow-up, about 30% of our data were censored. We took all-cause death as the end point of the study and did not exclude non cardiovascular death.

## Conclusion

Clinical characteristics, diagnosis and prognosis of AL-CA patients in southern China were examined. Although clinicians have improved their understanding of Al-CA in recent years, the prognosis of Al-CA is still poor, and the misdiagnosis rate and missed diagnosis rate are still very high in China. It is imperative to improve the recognition and early diagnosis of this condition, which may require multidisciplinary collaboration among cardiologists, hematologists and nephrologists.

## Supplementary Information

**Additional file 1.** Kaplan-Meier survival curves demonstrating differences in overall survival (months). The median survival time of AL-CA patients were diagnosed in the second half time (2016–2020) was longer than that of patients were diagnosed in the first half time (2012–2016) (12.00 vs 6.00, log-rank test, p = 0.35).

**Additional file 2.** Regional distribution of diagnosed patients with AL-CA in the present study.

**Additional file 3.** Median survival time of each subgroup.

**Additional file 4.** The diagnoses of 56 previously misdiagnosed patients with AL-CA.

## Data Availability

All data generated or analyzed during this study are included in this published article.

## Abbreviations

AL: Light-chain
CA: Cardiac amyloidosis
TTR: Transthyretin
HFpEF: Heart failure with preserved ejection fraction
ECG: Electrocardiographic
NYHA: New York heart association
eGFR: Estimated glomerular filtration rate
BMI: Body mass index
PRWP: Poor R-wave progression
CMR: Cardiac magnetic resonance
CHD: Coronary heart disease
MM: Multiple myeloma
COPD: Chronic obstructive pulmonary disease
CKD: Chronic kidney disease
SBP: Systolic blood pressure
DBP: Diastolic blood pressure
LMWH: Low molecular weight heparin
B + D: Bortezomib + dexamethasone
ASCT: Autologous stem cell transplantation
ThD/LeD: Thalidomide/lenalidomide
ALT: Alanine transaminase
AST: Aspartate aminotransferase
eGFR: estimated Glomerular Filtration Rate
CK-MB: Creatine kinase myocardial isoenzyme
CRP: C-reactive protein
LDL-C: Low density lipoprotein-cholesterol
TC: Total cholesterol
LDH: Lactate dehydrogenase
NT-proBNP: N-terminal pro–B-type natriuretic peptide
LVEDd: Left ventricular end diastolic diameter
RVEDd: Right ventricular end diastolic diameter
LAESd: Left atrium end systolic diameter
RAEDd: Right atrium end systolic diameter
IVS: Interventricular septum
LVPW: Left ventricular posterior wall
LVEF: Left ventricular ejection fraction
