# Supplementary material for: A real-world study on diagnosis and prognosis of light-chain cardiac amyloidosis in Southern China
Source: BMC Cardiovasc Disord. 2021 Sep 18;21:452. doi: 10.1186/s12872-021-02256-3 (PMC8449466; doi:10.1186/s12872-021-02256-3)
Supplement: Supplementary file 1 — Additional file 1. Kaplan-Meier survival curves demonstrating differences in overall survival (months). The median survival time of AL-CA patients were diagnosed in the second half time (2016–2020) was longer than that of patients were diagnosed in the first half time (2012–2016) (12.00 vs 6.00, log-rank test, p = 0.35). [file 12872_2021_2256_MOESM1_ESM.docx]

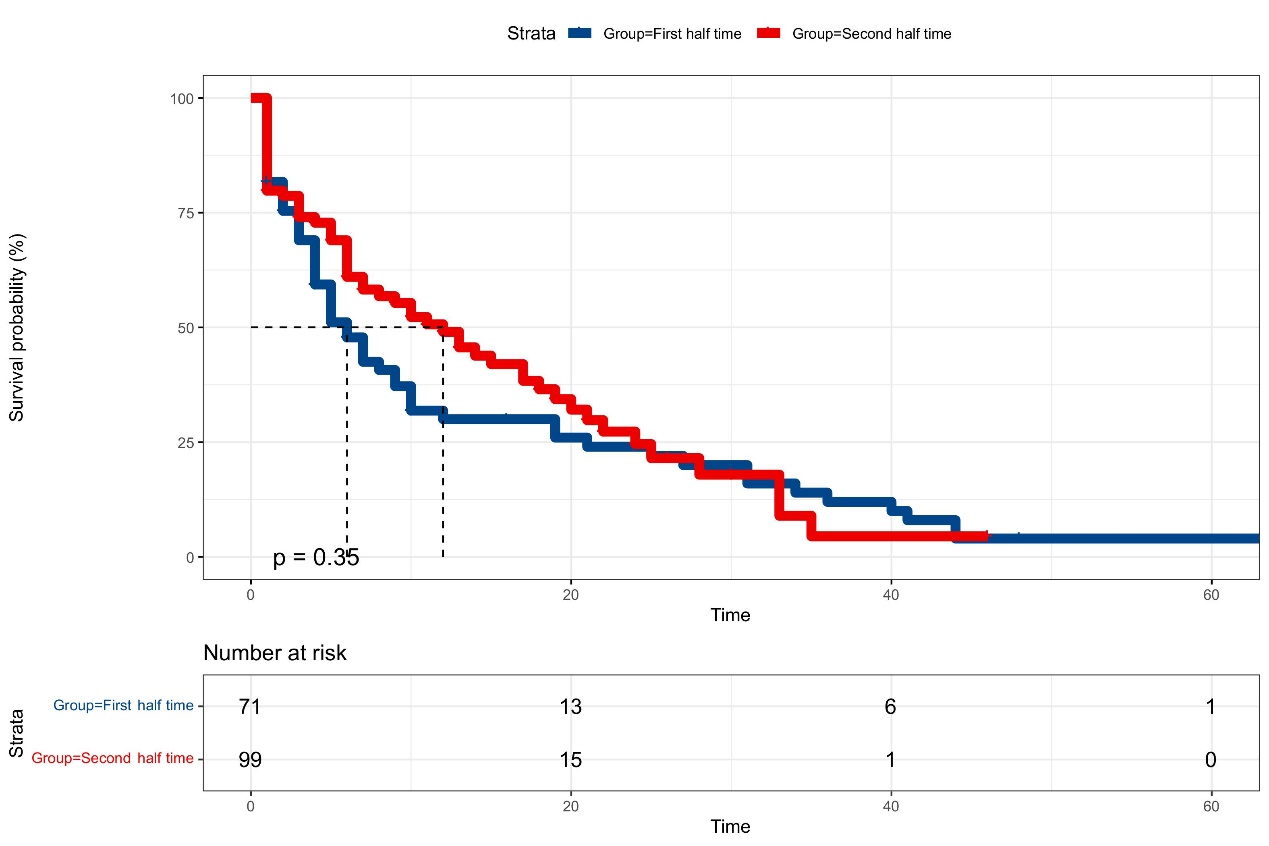


**Figure S1** Kaplan-Meier survival curves demonstrating differences in overall survival (months). The median survival time of Al-CA patients were diagnosed in the second half time was longer than that of patients were diagnosed in the first half time (12.00 vs 6.00, log-rank test, p=0.35).
