# Supplementary material for: A real-world study on diagnosis and prognosis of light-chain cardiac amyloidosis in Southern China
Source: BMC Cardiovasc Disord. 2021 Sep 18;21:452. doi: 10.1186/s12872-021-02256-3 (PMC8449466; doi:10.1186/s12872-021-02256-3)
Supplement: Supplementary file 2 — Additional file 2. Regional distribution of diagnosed patients with AL-CA in the present study. [file 12872_2021_2256_MOESM2_ESM.doc]

Table S1: Regional distribution of diagnosed patients with AL-CA in the present study.

| Provinces and cities | Number of people |
| --- | --- |
| Hunan | 145 |
| Changsha City | 40 |
| Hengyang City | 20 |
| Shaoyang City | 19 |
| Yongzhou City | 18 |
| Yueyang City | 14 |
| …… | …… |
| Guizhou | 5 |
| Jiangxi | 4 |
| Hubei | 4 |
| Sichuan | 4 |
| Guangdong | 3 |
| Fujian | 1 |
| Yunnan | 1 |
