# Supplementary material for: A real-world study on diagnosis and prognosis of light-chain cardiac amyloidosis in Southern China
Source: BMC Cardiovasc Disord. 2021 Sep 18;21:452. doi: 10.1186/s12872-021-02256-3 (PMC8449466; doi:10.1186/s12872-021-02256-3)
Supplement: Supplementary file 3 — Additional file 3. Median survival time of each subgroup. [file 12872_2021_2256_MOESM3_ESM.doc]

|  | Median survival time（months） | | 95%CI | P-value |
| --- | --- | --- | --- | --- |
| AL-CA | 8.00 | 5.63-10.37 | |  |
| AL-male | 9.00 | 6.54-11.46 | | 0.590 |
| AL-Female | 7.00 | 0.80-13.20 | |
| AL-NYHA I-II | 17.00 | 6.11-29.89 | | **0.006** |
| AL-NYHA III-IV | 6.00 | 4.67-7.53 | |
| AL-Chemotherapy | 13.00 | 4.49-21.51 | | **0.004** |
| AL-Palliative care | 6.00 | 3.36-8.64 | |
| AL-Chemotherapy（NYHA I-III） | 15.00 | 2.71-27.29 | | **0.031** |
| AL-Palliative care  （NYHA I-III） | 10.00 | 6.50-13.51 | |
| AL-MM | 7.00 | 3.75-10.25 | | 0.424 |
| Al-non-MM | 10.00 | 6.41-13.59 | |
| Al-high-eGFR^a^ | 9.00 | 6.23-11.78 | | 0.983 |
| Al-low-eGFR | 7.00 | 1.87-12.13 | |
| AL-HFrEF | 4.00 | 2.13-5.88 | | **0.001** |
| AL-HFpEF | 13.00 | 5.29-20.71 | |

Table S2. Median survival time of each subgroup

a：high eGFR=eGFR≥60mL/(min×1.73m2 )

MM：Multiple myeloma; NYHA: New York Heart Association Functional Classification;

For other abbreviations, see Table 1, 2 and 3.
