# Supplementary material for: A real-world study on diagnosis and prognosis of light-chain cardiac amyloidosis in Southern China
Source: BMC Cardiovasc Disord. 2021 Sep 18;21:452. doi: 10.1186/s12872-021-02256-3 (PMC8449466; doi:10.1186/s12872-021-02256-3)
Supplement: Supplementary file 4 — Additional file 4. The diagnoses of 56 previously misdiagnosed patients with AL-CA. [file 12872_2021_2256_MOESM4_ESM.docx]

Table S3 The diagnoses of 56 previously misdiagnosed patients with AL-CA

| Type of disease | Number of people (N, %) |
| --- | --- |
| Ischemic cardiomyopathy | 24 (42.9%) |
| Hypertrophic cardiomyopathy | 18 (32.1%) |
| Hypertensive cardiomyopathy | 10 (17.9%) |
| Other | 4 (7.1%) |
